# Nuclear retention of Fbw7 by specific inhibitors of nuclear export leads to Notch1 degradation in pancreatic cancer

**DOI:** 10.18632/oncotarget.1813

**Published:** 2014-03-21

**Authors:** Jiankun Gao, Asfar S. Azmi, Amro Aboukameel, Michael Kauffman, Sharon Shacham, Abdul-Badi Abou-Samra, Ramzi M. Mohammad

**Affiliations:** ^1^ Sichuan College of Traditional Chinese Medicine, Mianyang, Sichuan, People's Republic of China; ^2^ Department of Oncology, Karmanos Cancer Institute, Detroit, Michigan, USA; ^3^ Department of Pathology, School of Medicine, Wayne State University, Detroit, Michigan, USA; ^4^ Karyopharm Therapeutics, Natick MA, USA; ^5^ Hamad Medical Corporation, Doha, Qatar

**Keywords:** Specific Inhibitors of Nuclear Export, Xpo1, Exportin 1, CRM1 inhibitor, Notch1, Fbw7, Apoptosis, Pancreatic Cancer

## Abstract

Chromosome maintenance region 1 (CRM1) also called Exportin 1 (Xpo1), a protein found elevated in pancreatic ductal adenocarcinoma (PDAC), blocks tumor suppressor protein (TSP) function through constant nuclear export. Earlier we had shown that targeting CRM1 by our newly developed specific inhibitors of nuclear export (SINE) leads to inhibition of pancreatic cancer cell proliferation and tumor growth arrest. In this paper we define the mechanism of SINE action. Our lead SINE KPT-185 inhibits PDAC cell growth, cell migration, tumor invasion and induces apoptosis and G2-M cell cycle arrest in low nano molar range (IC_50s_~150 nM). Mechanistically we demonstrate that the activity of KPT-185 is associated with nuclear retention of Fbw7; which degrades nuclear Notch-1 leading to decreased tumor promoting markers such as C-Myc, Cyclin-D1, Hes1 and VEGF. The orally bioavailable SINE (KPT-251) showed potent anti-tumor activity in a Colo-357 PDAC xenografts model; residual tumor analysis showed activation of Fbw7 concomitant with attenuation of Notch1 and its downstream genes. These results suggest that the antitumor activity of KPT-185 is in part due to nuclear retention of Fbw7 and consequent Notch1 degradation. The new CRM1 inhibitors, therefore, hold strong potential and warrant further clinical investigations for PDAC.

## INTRODUCTION

Pancreatic Ductal Adenocarcinoma (PDAC) is the fourth deadliest disease that accounts for ~38,000 annual deaths in the United States [1], suggesting that newer and effective treatment strategies are critically needed. The majority of PDAC patients become refractory to standard chemotherapeutic drugs such as gemcitabine and 5FU or their combination [2]. In principle, most chemotherapeutic drugs work through activation and nuclear localization of tumor suppressor proteins (TSPs) such as p53, FOXO, p27 and IkB. However, in PDAC and other cancers, aberrant over-expression of nuclear exporter protein CRM1/Exportin 1/Xpo1 results in mis-localization of TSPs thereby inhibiting their cell surveillance activity [3,4]. Therefore, blocking nuclear export machinery could become a viable therapeutic strategy to restore tumor suppressor functions as well as the apoptosis machinery [5].

Over-expression of nuclear Notch1 mediated signaling in PDAC has been well established [6,7]. Notch1 carries a large single-pass type 1 trans-membrane receptor that is activated by interaction with membrane bound ligands [8]. For activation Notch1 receptors undergo series of proteolytic cleavage resulting in the activation of Notch1 intracellular domain (Notch1-IC) that translocates into the nucleus [9]. The active forms of Notch1, along with other transcription factors, regulate the expression of many tumor promoting genes [10]. Fbw7 (also known as Fbxw7, SEL-10, hCdc4, or hAgo) is the F-box protein subunit of a Skp1-Cul1-F-box protein (SCF)-type ubiquitin ligase complex that plays an important role in the degradation of Notch family members including the activated Notch1C in the nucleus [11,12]. Nevertheless, mislocalization of Fbw7 by CRM1 over-expression results in Notch1 nuclear accumulation and consequent activation of tumor promoting pathways. Therefore, we hypothesize that CRM1 inhibition could potentially restore nuclear Fbw7 leading to degradation of nuclear Notch1 thereby inducing PDAC cell death.

**Figure 1:**
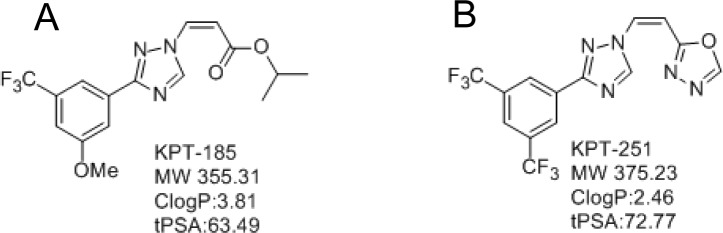
Structures of A KPT-185 and B. KPT-251

Using structure based drug design, we have recently developed specific inhibitors of nuclear export (SINE Fig 1A & B). NCI 60 cell line screening shows that the inhibitors have broad tumor specificity and low nano molar potency. Recently, we showed that SINE can suppress proliferation of PDAC [13] and NHL [14] cell lines and induce tumor growth arrest in their corresponding sub-cutaneous and orthotopic tumor models. Most significantly, SINEs do not induce growth inhibition and apoptosis in normal peripheral lymphocytes, NIH-3T3 cells or mouse fibroblast. Additionally, SINEs have excellent pharmacokinetic parameters, have passed advanced toxicity profiling and are currently in phase I clinical trials for solid tumors and hematological malignancies. Using these promising inhibitors, in this study, we investigated their mechanism of action in a panel of PDAC lines and animal tumor model explained in the light of Fbw7-Notch.

## RESULTS

### Effects of KPT-185 on the viability of pancreatic cancer cells

**Figure 2:**
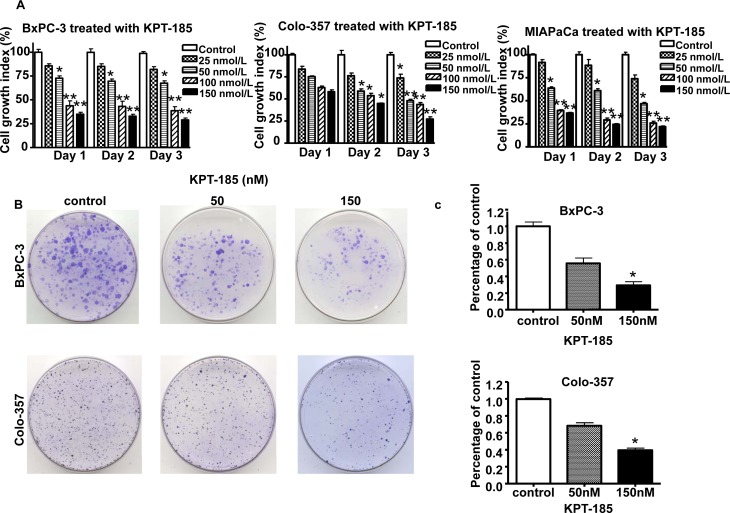
Effects of KPT-185 on PC cell growth A. BxPC-3, Colo-357 and MIAPaCa cells were seeded in 96-well plates at a density of 5,000 cells per well and treated with the indicated concentrations of KPT-185 for 0-72 hrs. After treatment, cell viability was determined using the MTT assay as described in Materials and Methods. Vertical bars indicate the means ± SD of three independent experiments. *p<0.05, **p<0.01 versus control. B. Cells treated with different concentrations of KPT-185 for 72 h were evaluated by the clonogenic assay. Photographic difference in colony formation in BxPC-3 and Colo-357 untreated and treated with KPT-185 are shown. C. Quantification of colony numbers in control vs treated BxPC-3 and Colo-357 cells shown in B. * *p* values represent comparisons between cells treated by KPT-185 and control using the paired *t* test.

In order to investigate the effect of KPT-185 on cell growth, we evaluated the cell viability of PDAC cells treated with KPT-185 for 72 hrs using the MTT assay. As presented in Figure 2 A. KPT-185 inhibits cell growth in a dose dependent manner in all the three cell lines (IC_50s_ ~150 nM). To confirm the effects of KPT-185 on cell growth, clonogenic assay was performed. Fig 2 B and C show a dose dependent inhibition of clonogenic potential by KPT-185 in Colo-357 and BxPC-3 cells. The results from the clonogenic assay were consistent with the MTT data shown suggesting that KPT-185 inhibited cell growth in BxPC-3, Colo-357 PDAC cells.

### Inhibition of cell migration and invasion by KPT-185

**Figure 3:**
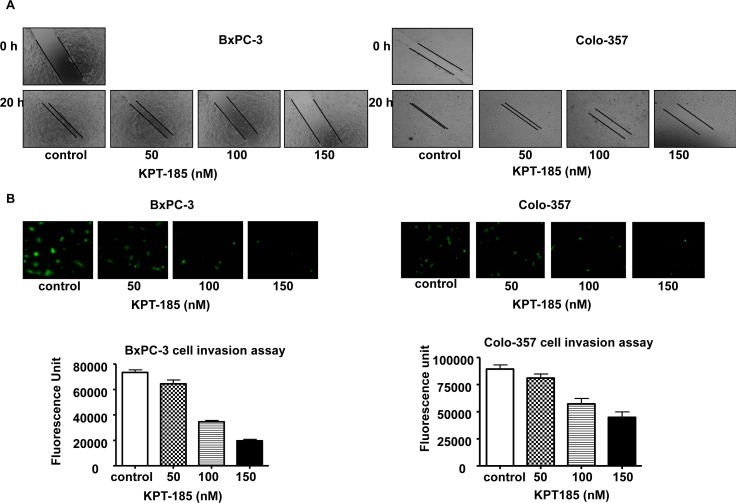
KPT-185 inhibits PC cell migration and invasion A Dose-dependent inhibition of PC cell migration was observed by KPT-185 using the wound healing assay. The wound was generated in the cells with 90-95% confluent by scratching the surface of the plates with a sterile pipette tip. The cells were then incubated in the absence and presence of KPT-185 for 20 h; wound healing images were captured by a Nikon microscope. B. Dose-dependent inhibition of PC cells invasion by KPT-185. Cells that invaded to the lower surface of the insert over a period of 20 h were stained with calcein AM. The fluorescently labeled invasive cells were photographed by a fluorescent microscope. The fluorescence of the invaded cells was read in ULTRA Multifunctional Microplate Reader (TECAN, Switzerland) at excitation/emission wavelengths of 485/530 nm. Columns, mean; bars, SD.

Notch over-expressing cells such as BxPC-3 and Colo-357 demonstrate high proliferation and migratory properties. In order to examine whether KPT-185 can prevent their migratory and invasive potential, we conducted wound healing and invasion assays. As shown in Fig. 3 A, KPT-185 inhibited cell migration in a dose dependent manner in both BxPC-3 and Colo-357 PDAC cells. The results of Fig. 3 B clearly show that KPT-185 treated BxPC-3 and Colo-357 cells decrease in their invasive capability as compared to the untreated control. These results confirm that KPT-185 can inhibit cell migration and invasion and whether this results in cell growth arrest was investigated as shown below.

### KPT-185 induces cell cycle arrest in PDAC cells

**Figure 4:**
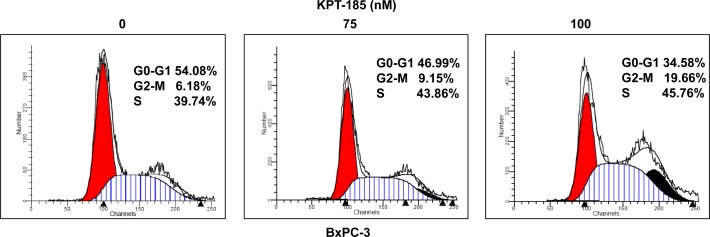
KPT-185 induces cell cycle arrest at G2-M phase BxPC-3 cells were exposed to the indicated concentrations (0, 75, 100 nM) of KPT-185 for 72 hrs and cells were harvested for cell cycle analysis using propidium iodide staining. X axis, DNA content; Y axis, number of nuclei.

In order to verify whether KTP-185 works by abrogating cell cycle progression, propidium iodide flow cytometry assays were performed. BxPC-3 cells were exposed to 75 and 100 nM of KPT-185 for 72 hrs and analyzed for cell cycle distribution. As expected, increasing concentrations of KPT-185 resulted in G2-M arrest patterns (Fig 4). Similar results were obtained for Colo-357 cells (data not shown). These findings further solidify the case for KPT-SINEs as effect agents against PDAC. We further investigated the apoptotic potential and also evaluated the mechanism supporting KPT-185 activity and the results are presented below.

### KPT-185 induces apoptosis in PDAC cell lines

**Figure 5:**
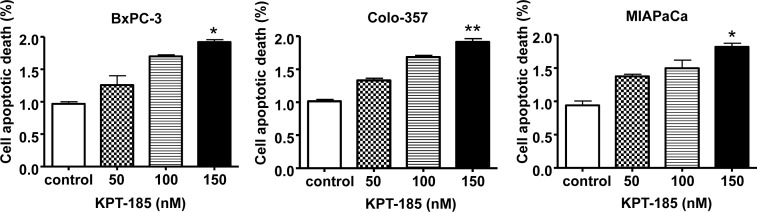
KPT-185 induces apoptosis in PC cells BxPC-3, Colo-357 and MIAPaCa cells were exposed to different concentrations of KPT-185 for 72 h. Apoptosis were determined by histone/DNA ELISA. Columns, mean; bars, SD. *p<0.05, **p<0.01, compared with control.

In order to verify that growth inhibition by KPT-185 in PDAC is due to induction of apoptosis Histone DNA ELISA assays were performed. Histone DNA ELISA is a highly sensitive assay that quantitatively measures apoptotic cell death. As demonstrated in Fig. 5 and in line with the MTT and colonogenic assays, increasing concentrations of KPT-185 induced a progressive increase in apoptosis in three PC cell lines BxPC-3 (Fig. 5 A), MIAPaCa (Fig. 5 B) and Colo-357 (Fig. 5 C). These results confirm that KPT-185 is a potent apoptosis inducer in PC and we further sought to investigate its mechanism of action as presented below.

### KPT-185 targets Fbw7-Notch Axis in PDAC

**Figure 6:**
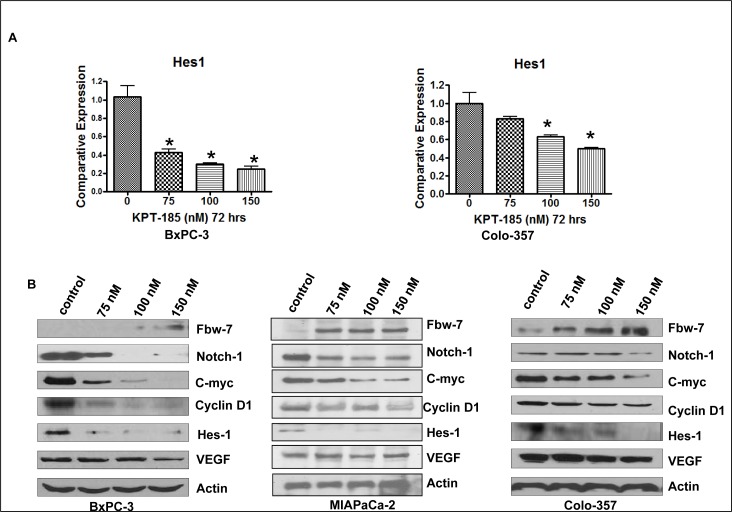
KPT-185 Targets Fbw7-Notch axis in PC A. The mRNA levels of Hes-1 was investigated by real-time RT-PCR in BxPC-3 and Colo-357 PC cells treated with KPT-185 for 72 h. Columns, mean; bars, SD. *p<0.05, **p<0.01, compared with the control. B. Nuclear protein levels of Fbw7, Notch1, C-myc, CyclinD1, Hes-1 and VEGF detected by Western blotting in BxPC-3 and Colo-357 PC cells treated with KPT-185 (0-150 nM) for 72 hr.

To further understand the molecular mechanism involved in KPT-185 induced cell growth inhibition, cell cycle arrest and apoptosis, alterations in the cell survival pathway were investigated using Western blot analysis with especial emphasis on Notch1 signaling. BxPC-3 and Colo-357 cell lines treated with KPT-185 for 72 hrs were assessed using real-time reverse transcription-PCR (RT-PCR) analysis. As shown in Fig. 6 A, the mRNA expression of Hes1 gene decreased after KPT-185 treatment in both cell lines. Additionally the effect of KPT-185 on Fbw7 nuclear export in PC cells was assessed by subjecting nuclear extracts from KPT-185 treated BxPC-3 and Colo-357 cells to Western blotting. As shown in Fig. 6 B, compared to control, KPT-185 treatment (0-150 nM), leads to significantly enhanced accumulation of Fbw7 with concomitant down-regulation of Notch1 protein. Most significantly, we also observed down-regulation of Notch pathway proteins (Hes-1, C-myc, VEGF and CyclinD). These results solidify our hypothesis that KPT-185 works through nuclear retention of the Notch suppressor Fbw7. Based on these strong *in vitro* findings we investigated the *in vivo* potential of analog KPT-251 in xenograft developed from Colo-357 cell lines.

### Pre-clinical efficacy trial of KPT-SINE

**Figure 7:**
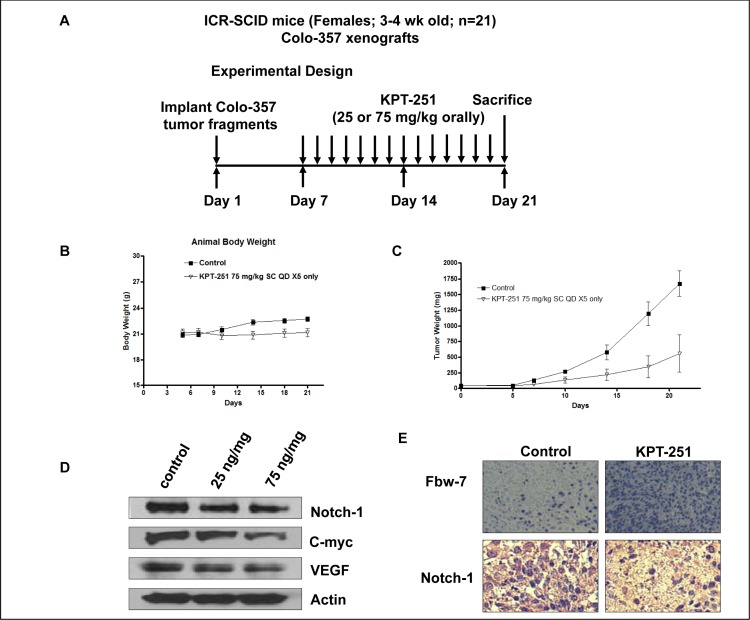
In vivo anti-tumor efficacy and molecular mechanism of action of KPT-251 A. Schematic representation of the experimental design. B. KPT-251 treated ICR SCID animal body weight loss evaluation. Note: no statistically significant loss in body weight on KPT-251 treatment. The MTD was determined to be 75 mg/kg sc. C. Efficacy trial of KPT-251 treated at 75mg/kg bw daily for 2 weeks. Note KPT-251 treatment drastically reduced tumor growth in comparison to control. D. The expression of Notch1, C-myc and VEGF was detected by Western blotting of tumor tissue extracts. E. immunohistochemical staining of Colo-357 tumor xenografts. Tumors were resected and processed for routine histologic analysis and the 5 μmol/L tissue sections were stained with antibodies to Notch1 and Fbw7.

For *in vivo* studies we challenged SCID mice carrying Colo-357 sc tumors with the orally available analog KPT-251 (Fig 7 A for schema of treatment). SINEs have been developed as safe anti-tumor agents for cancer therapy and do not show toxicity. Body weight of the host animal remains stable during the course of treatment with the Maximum tolerated dose (MTD) of KPT-251, calculated to be 75 mg/kg sc for 20 days, (Fig 7 B). Most significantly, and in line with the *in vitro* results, oral administration of KPT-251 at 75 mg/kg resulted in drastic tumor regression in the Colo-357 animal models (Fig 7 C). To further investigate whether KPT-251 down-regulates Notch1 *in vivo*, we examined Notch1 expression in tumor tissues obtained from KPT-251-treated animals. As expected, Western blot analyses of animal tissues demonstrated significant down-regulation of Notch1 and its related proteins (c-myc and VEGF Fig 7 D). Interestingly, we observed induction of Fbw7 and suppression of Notch1 in KPT-251 treated tumors (Fig. 7 E). These data show that KPT holds strong anti-tumor potential and warrant further clinical application for the treatment of PDAC.

## DISCUSSION

This study shows that CRM1 inhibition and consequent nuclear retention of tumor suppressor proteins by specific inhibitors of nuclear export could become a therapeutic strategy for PDAC. Mechanistically we have discovered that our CRM1 inhibitors work through a novel Fbw7 nuclear retention mediated notch1 suppressive mechanism that could be replicated both *in vitro* and *in vivo*. Based on these strong pre-clinical findings, we propose that our novel CRM1 inhibitors hold strong translational potential and warrant further clinical investigations in PDAC.

The majority of PC patients are intractable to currently available treatment modalities [15]. A major reason for failure chemotherapeutic agents such as gemcitabine, platinum based drugs and 5FU has been attributed to inadequate activation of tumor suppressor proteins [16]. In instances where TSPs are activated, their function is inhibited by mislocalization through constant nuclear export by CRM1 protein [17]. CRM1 is the major TSPs and its importance can be gauged by the observation that the protein is found over-expressed in most cancers including PDAC [3]. Therefore, targeted inhibition of the nuclear exporter CRM1 becomes an attractive therapeutic strategy for the treatment of PC. Earlier attempts to target CRM1 led to development of agents such as Leptomycin B (LMB), although specific inhibitors of CRM1, yet showed adverse off target toxicities [18].

To overcome toxicity, we have developed novel highly specific non-toxic small molecules capable of antagonizing the functions of CRM1 [19]. Unlike, Leptomycin B that form an irreversible covalent bond with Cys528 of CRM1, KPT-185 bind to CRM1 in a slowly reversible fashion, which might contribute to the improved tolerability of the SINE compounds. SINE block nuclear export of TSPs thereby inducing growth inhibition and apoptosis specifically in cancer cells [20,21,22,23,24,25,26,27,28]. Earlier, our group has studied the impact of CRM1 inhibtion in PDAC models and the downstream signaling analysis [13]. These studies deciphered the downstream targets including FOXO3a, p27, p21 and the pro-apoptotic protein prostate apoptosis response 4 (PAR-4) mediated effects both in vitro as well as in animal models of PDAC. In the current study, we investigated the anti-tumor potential and molecular mechanism of action by which our CRM1 inhibitors elicit their biological effects in PDAC cells and tumor models. The lead CRM1 inhibitor KPT-185 effectively caused growth inhibition, suppressed colonogenic potential and induced G2-M cell cycle arrest and apoptosis in a panel of PDAC cell lines. As such these investigations proved the potential of KPT-185 as new anti-cancer agents for PDAC.

The TSP Fbw7, among the nuclear export targets of CRM1, plays a central role in the degradation of Notch family members. Fbw7 has been recently shown to exert its anti-tumor effects through degradation of Notch1 [29]. Some reports have shown that Fbw7 binds to phosphorylated Notch 1C and mediates its ubiquitination and subsequent rapid degradation [30]. Additionally, Fbw7 exerts its inhibitory effect by regulating Notch1 downstream signaling pathways through ubiquitin ligase mediated degradation [31]. However, there are no available agents that enhance FBW7 or induce its nuclear retention. These key observations led to our hypothesis that targeted inhibition of CRM1 and nuclear retention of Fbw7 could become a viable therapeutic strategy for PDAC. In line with our hypothesis we observed that treatment with KPT-185 resulted in nuclear accumulation of Fbw7 with consequent down-regulation of Notch1 and related pathways (Hes-1, C-Myc and VEGF) in PDAC cells and this was correlated with our growth inhibition, cell cycle arrest and apoptosis data.

Prior to clinical application of a new therapeutic agent, such as SINEs, pre-clinical evaluation in a suitable animal model is required. Therefore, we tested the *in vivo* potential of the drugs in xenograft models derived from Colo-357 cells that are recognized to over-express Notch. In line with our *in vitro* growth inhibition and apoptosis results, the analog KPT-251 showed remarkable anti-tumor activity in the Colo-357 model and most importantly had no visible toxicity to host animals. Of paramount significance is the observation that in residual tumors we could replicate the *in vitro* mechanistic findings and observed clear activation of Fbw7 and suppression of Notch signaling.

In summary, we have provided experimental evidence that supports the role of SINEs as antitumor agent for PDAC. Mechanistically, we propose that our SINEs attenuate Notch1 signaling, cell proliferation, invasion and induce apoptosis in PC cells by degradation of Notch1 through nuclear retention of Fbw7 (Synopsis graphical abstract for summary mechanistic schema). However, further studies are needed to confirm the molecular regulation of Notch1 by SINE induced-Fbw7 expression prior to their clinical application for PDAC.

**Figure 8:**
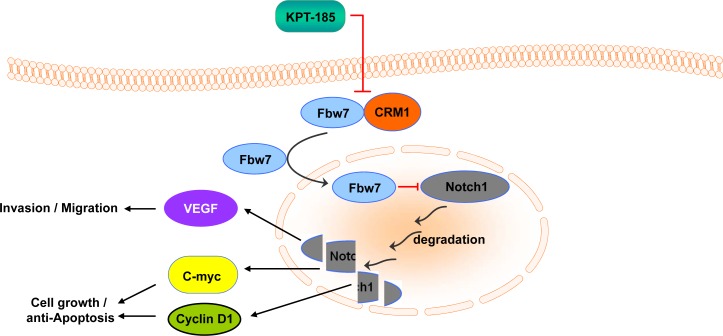
Summary Scheme Chromosome maintenance region 1 (CRM1) is the major nuclear exporter of tumor suppressor proteins (TSPs) and has been considered as a therapeutic target in cancer. We have developed a specific inhibitor of nuclear export (SINE) that blocks CRM1 leading to restoration of important TSPs in cancer cell nucleus. Mechanistically, we show that our SINEs attenuate Notch1 signaling, cell proliferation, invasion and induce apoptosis in a pancreatic cancer model by degradation of Notch1 through nuclear retention of Fbw7. The schematic representation of the proposed molecular mechanism of SINE induced pancreatric cancer cell growth inhibition and apoptosis.

## MATERIALS AND METHODS

### Cell culture and experiment reagents

Human PDAC cell lines BxPC-3, Colo-357 and MIAPaCa used in this study were obtained from ATCC and maintained in standard cell culture conditions (at 37 °C in a humidified 5% CO_2_ atmosphere). Primary antibody for Notch1, CyclinD1, VEGF were purchased from Santa Cruz Biotechnology (Santa Cruz, CA), Fbw7 and Hes-1 were purchased from the Abcam (San Francisco, CA), C-myc, Bcl-2 were purchased from Cell signaling (Danvers, MA). The secondary antibodies were bought from Sigma (St. Louis, MO). SINEs (KPT-185 and KPT-251) were designed, synthesized and purified by Karyopharm Therapeutic (Natick, MA).

### Cell viability Studies by MTT assay

The BxPC-3, Colo-357 and MIAPaCa cells (5×10^3^) were seeded in a 96-well culture plate. 24 hrs after seeding, cells were treated with various concentrations of most potent KPT-SINE KPT-185 for 24, 48, and 72 hrs. At the end of treatment period, 10 uL of Reagent MTT (Sigma Chemical Company 5 mg/mL in PBS) was added to each well. After 2 hrs incubation 100 μL of Isopropanol was added to each well and further incubated for 20 minutes in dark. The color intensity was measured by TECAN's microplate fluorometer (TECAN Switzerland) at 595 nm.

### Clonogenic assay

To test the survival of cells treated with KPT-185, BxPC-3 and Colo-357 cells were plated (3×10^5^ per well) in a six-well plate and incubated overnight. After 72 hrs exposure to various concentrations of KPT-185, the viable cells were counted and plated in 100 mm dishes in a range of 3,000 cells per plate. The cells were then incubated for 21 days at 37 °C in a humidified 5% CO_2_ atmosphere. All the colonies were fixed in 4% Paraformaldehyde and stained with 2% crystal violet.

### Wound healing assay

Wound healing assay was conducted to examine the capacity of cell migration. BxPC-3 and Colo-357 were seeded in a six well plate at the concentration of 3×10^5^ cells per well. The wound was generated in the cells with 90-95% confluent by scratching the surface of the plates with a sterile pipette tip. The cells were then incubated in the absence and presence of KPT-185 for 20 hrs, and then photographed with a Nikon microscope.

### Cell invasion assay

BD Biocoat invasion kit (BD, San Jose, CA) was used to evaluate the tumor invasive ability. Briefly, around 2.5×10^4^ cells of BxPC-3 and Colo-357 with basal media was transferred in each 6-well upper chamber in the presence or absence of KPT-185. 0.75 milliliter of culture medium with 5% FBS was added into each bottom chamber of 6-well plate. After 20 hrs of incubation, the cells in the upper chamber were removed, and the cells that had invaded through Matrigel matrix membrane were stained with 4 μg/mL calcein AM in PBS at 37 °C, 5% CO_2_ for 1 hr. The fluorescence of the invaded cells was read in ULTRA Multifunctional Microplate Reader (TECAN, Switzerland) at excitation/emission wavelengths of 485/530 nm. These fluorescently labeled invasive cells were also photographed under a fluorescent microscope.

### Flow cytometry and cell cycle analysis

Cells were seeded in 100 mm dish per plate, incubated overnight. Subsequently, all the cells were serum starved for another 24 hrs. The cells were exposed vehicle (DMSO) or KPT-185 and grown for 72 hr. At the end of treatment period, cells were collected and fixed with ice-cold 70% (v/v) ethanol for 24 hr. After centrifugation at 3000×g for 5 min, the cell pellets were washed with PBS (pH 7.4) and resuspended in PBS containing propidium iodide (50 μg/mL), and DNase-free RNase A (100 μg/mL). Samples were then incubated at 37 °C for 15 min, and DNA contents were determined by flow cytometry using a flow cytometer (BD, San Jose, CA) by the Karmanos Cancer Institute Flow cytometry core.

### Histone/DNA ELISA for detection of apoptosis

The Cell Death Detection ELISA Kit (Roche Applied Science, Indianapolis, IN) was used to detect apoptosis in pancreatic cells. 3×10^5^ cells were seeded in six-well plates. After 24 hrs incubation, cells were treated with various concentrations of KPT-185 for 72 hrs. The cells were then lysed, and cytoplasmic histone/DNA fragments were extracted and incubated in microtiter plate modules coated with anti-histone antibody. In order to detect the immobilized histone/DNA fragment, Peroxidase-conjugated anti-DNA antibody was used before color development with ABTS substrate for peroxidase. The spectrophotometric absorbance of the sample was determined by TECAN's microplate fluorometer (TECAN, Switzerland) at 405 nm.

### Protein extraction and western blotting

For protein extraction, KPT-185 treated cells were sonicated in 125 mM Tris-HCl and 4% SDS according to our previously published methods [32]. In another set of experiments, cytoplasmic and nuclear proteins were also extracted using NE-PER Nuclear and Cytoplasmic Extraction Reagents (Thermo Scientific, Rockford, IL). The protein concentrations were determined using the BCA™ protein assay reagent (Thermo Scientific, Rockford, IL). Proteins were fractionated using sodium dodecyl sulfate-polyacrylamide gel electrophoresis (SDS-PAGE), and the gels were transferred onto nitrocellulose membrane. The membranes were blocked with 4% nonfat dried milk or bovine serum albumin in 1×PBS containing 0.1% Tween-20 and then incubated over night at 4 °C with appropriate primary antibodies. The membranes were washed 3 times with PBS-T, and subsequently incubated with the secondary antibodies for 2 hrs at room temperature. The protein bands were detected using the enhanced chemiluminesence detection system (Genscript, Piscataway, NJ).

### Real-time quantitative PCR for gene expression analysis

The total RNA was isolated by Trizol (Invitrogen, Carlsbad, CA) according to the manufacturer's protocols. Two microgram of total RNA from each sample was subjected to first strand cDNA synthesis using High capacity RNA to cDNA master mix (Life Technologies Corporation, CA) in a total volume of 20 μL. Reverse transcription reaction were performed at 37 °C for 1 h, followed 95 °C for 5 min. Real time PCR was used to quantify mRNA expression by using SYBR^®^ Green RT-PCR Reagents (Life Technologies Corporation, CA). Sequences of primers were sets used for this analysis are as follows: Hes-1, forward (- AACACGACACCGGATAAACC -3') and reverse primer (5'- CCGCGAGCTATCTTTCTTCA -3'); GAPDH, forward (5'-ACCCAGAAGACTGTGGATGG-3') and reverse primer (5'-CAGTGAGCTTCCCGTTCAG-3'). The PCR were performed in a total of 10 μL reaction mixture (2 μL of cDNA, 17 μL of 2× SYBR Green PCR Mix, 1.4 μL of each 5 μmol/L forward and reverse primers, and 13.6 μL of Nuclease-free water for 3 well) in StepOne Plus Real-Time PCR System (Applied Biosystems, CA). The PCR program was initiated by 10 minutes at 95°C before 45 thermal cycles, each for 15 seconds at 95°C and 1 minute at 60°C. Data were analyzed according to the comparative fold increase or decrease in gene expression determined by ct values and normalized by GAPDH expression in each sample.

### Colo-357 xenografts

Four-week-old female ICR-SCID mice were obtained from Taconic Laboratory and xenografts were developed by injecting 10^7^ Colo-357 cells s.c. in each flank area. When s.c. tumors developed to about 1,500 mg, the tumors were excised, and serial propagation was accomplished by trimming extraneous material, cutting the tumors into fragments of 50 mg, which were then transplanted s.c. into the flanks of a new group of mice for maintenance of tumors as well as for experimental purpose. For the subsequent drug efficacy trials, small fragments of the Colo-357 xenograft were implanted s.c. and bilaterally into naive, similarly adapted mice (n=20). Mice were checked 3 times per week for tumor development. Once transplanted, Colo-357 fragments developed into palpable tumors (60–100 mg); animals were removed randomly and assigned to different treatment groups (10 per group). Using this model, the efficacy of KPT-251 (analog of KPT-185, used *in vivo*) was studied. The maximum tolerated dose of KPT-251 in severe combined immunodeficient mice was determined to be 75 mg/kg. Mice were administered with KPT-251 at 25 or 75 mg/kg orally, for 2 weeks. (as shown in Fig 6A.). Mice in the control and KPT-251 treated group were followed for measurement of s.c. tumors, changes in body weight and side effects of the drugs. Tumor was calculated using the formula (A ×B^2^)/2, where A and B are the tumor length and width (in mm). To avoid discomfort in the control group, animals were euthanized when their total tumor burden reached 2,000 mg. Tumor tissues harvested from this experiment were used for immunohistochemical and Western blotting analyses. All studies involving mice were performed under Animal Investigation Committee approved protocols at Wayne State University.

### Immunohistochemical determination of Notch1 and Fbw7

The expression of Notch1 and Fbw7 was detected in histologic sections of tumor xenografts. Sections were cut from formalin-fixed, paraffin-embedded tissue blocks; collected on 3-ethoxy-aminoethyl-silane-treated slides; and allowed to dry overnight at 37 °C. Sections were dewaxed in xylene, rehydrated through graded concentrations of ethanol to distilled water, immersed in 10 mmol/L citrate buffer (pH 6.0), and processed in a thermostatic water bath for 40 minutes at 98°C for antigen retrieval. Anti-Notch1, and anti-Fbw7 antibodies were applied on two slides for each case, and incubations were done overnight at room temperature in a humidified atmosphere followed by a 30-minute incubation of secondary antibody. Slides were then incubated with streptavidin peroxidase and visualized using the 3,3'-diaminobenzidine chromogen (Lab Vision Corp., Fremont, CA).

### Tumor Tissue proteins isolation and western blot analysis:

At the end of the treatment period tumors were excised and one part was minced for protein isolation according to our previously published methods. 100 μg tumor protein lysates were resolved using western blotting assay. The membranes were probed for Notch1, C-myc, VEGF and β-actin.

Data Analysis: Data are represented as mean ± SD for the absolute values or percentage of controls as indicated in the vertical axis legend of figures. The statistic significance of differential findings between experimental groups and control groups was statistically evaluated ANOVA using GraphPad StatMate software (GraphPad Software, Inc., San Diego, CA). *p* values lower than 0.05 were considered statistically significant.
